# Improving Lifelong Comprehensive Care Coordination in Nephropathic Cystinosis: Multidisciplinary Perspectives

**DOI:** 10.1016/j.ekir.2025.103735

**Published:** 2025-12-18

**Authors:** Ladan Golestaneh, Elizabeth G. Ames, Maya H. Doyle, Cybele Ghossein, Paul C. Grimm, Jennifer L. Hewlett, Alaena Lim, Lauren S. Marzinelli, Kimberly J. Schmidt, Bonnie Smeryage, Joshua J. Zaritsky, Frederick J. Kaskel

**Affiliations:** 1 Department of Internal Medicine, Section of Nephrology, Yale School of Medicine, New Haven, Connecticut, USA; 2 Department of Pediatrics, Division of Pediatric Genetics, Metabolism & Genomic Medicine, and Department of Internal Medicine, Division of Genetic Medicine, Michigan Medicine, Ann Arbor, Michigan, USA; 3 Department of Social Work, School of Health Sciences, Quinnipiac University, Hamden, Connecticut, USA; 4 Division of Pediatric Nephrology, Children’s Hospital at Montefiore, Bronx, New York, USA; 5 Department of Medicine, Division of Nephrology & Hypertension, Feinberg School of Medicine, Northwestern University, Chicago, Illinois, USA; 6 Department of Pediatrics, Division of Nephrology, Stanford Medicine, Palo Alto, California, USA; 7 Department of Pharmacy, Children’s Hospital of Philadelphia, Philadelphia, Pennsylvania, USA; 8 Department of Human Genetics, Emory School of Medicine, Atlanta, Georgia, USA; 9 Division of Nephrology & Hypertension, Northwestern Medicine, Chicago, Illinois, USA; 10 Genetics and Metabolic Clinic, St. Luke’s Health System, Boise, Idaho, USA; 11 Division of Pediatric Nephrology, Joe DiMaggio Children’s Hospital, Hollywood, Florida, USA; 12 Division of Nephrology, Phoenix Children’s Hospital, Phoenix, Arizona, USA; 13 Department of Pediatrics (Pediatric Nephrology), Albert Einstein College of Medicine, Bronx, New York, USA

**Keywords:** care coordination, cystinosis, integrated care, lysosomal storage disorder, multidisciplinary care, rare disease

## Abstract

Nephropathic cystinosis is a rare lysosomal storage disorder characterized by cellular cystine accumulation and progressive multiorgan damage. Before advances in diagnostics, transplantation, and disease-modifying therapy, cystinosis was considered a fatal pediatric disease, with most patients reaching kidney failure by approximately 10 years of age. Life expectancy has now expanded into the 50s and beyond, and the disease has been transformed into a chronic condition with a predominantly extrarenal phenotype in adulthood. Traditionally, nephrologists have played a central role as cystinosis “care quarterbacks” in pediatric settings, and patients often expect this to continue after transitioning to adult care. As such, nephrologists are increasingly tasked with monitoring for and addressing complications in organs other than the kidneys, in addition to coordinating referrals to clinicians within the nephrology clinic (e.g., nurses, advanced practice providers, pharmacists, dietitians, social workers, care coordinators, and transplant specialists) and across multiple specialties (e.g., neurologists, gastroenterologists, endocrinologists, ophthalmologists, and orthopedists). Although recently published expert guidance offers recommendations for the multidisciplinary management of cystinosis, gaps in the literature exist around strategies to reduce care fragmentation, overcome collaboration challenges, and enhance patient/family and clinician experiences. This review aimed to explore various approaches for care delivery optimization across the lifespan of patients with cystinosis as well as improve the current understanding of disease-, patient-, provider-, and system-related factors that influence treatment adherence, engagement, and long-term outcomes.

Cystinosis is a rare disease caused by biallelic variants in the *CTNS* gene that encodes the lysosomal transporter cystinosin.^1^^,^^2^ *CTNS* is expressed in all tissues, and absent or defective cystinosin results in cellular accumulation of the amino acid cystine, to levels 50 to 100 times above normal, leading to progressive multiorgan damage and dysfunction.^3^, ^4^, ^5^ This lysosomal storage disorder follows an autosomal recessive inheritance pattern and has an approximate global incidence of 1 in 100,000 to 200,000 live births, although this figure varies widely by geographic location and is likely underestimated (Figure 1).^2^^,^^6^

**Figure 1:**
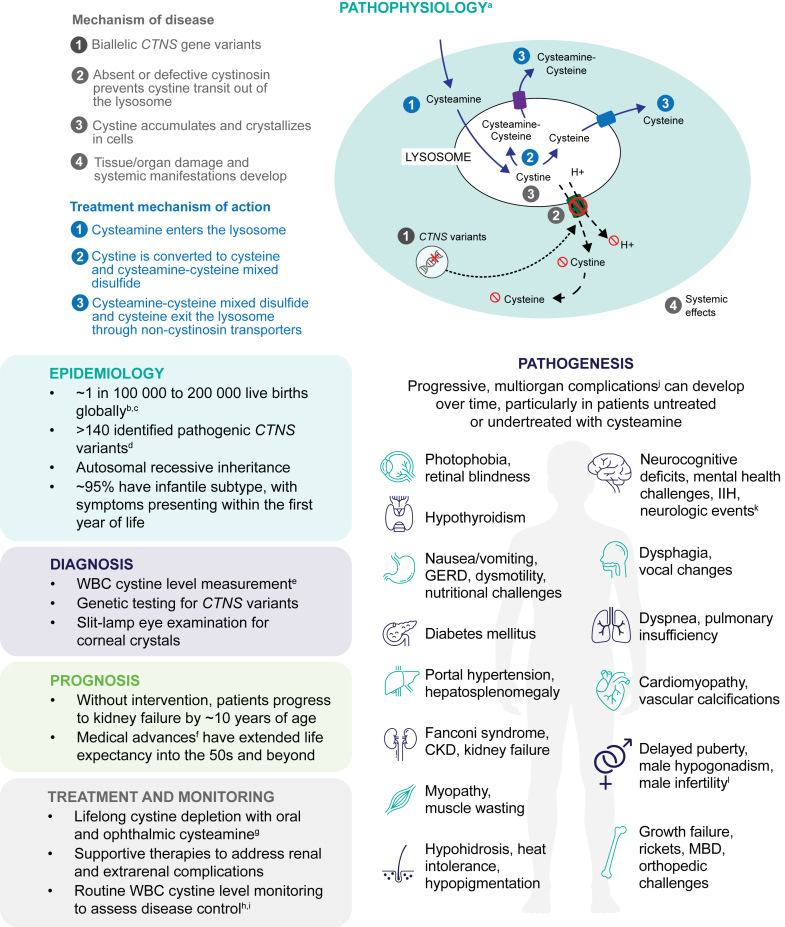
Overview of nephropathic cystinosis, a rare lysosomal storage disorder characterized by cellular cystine accumulation and progressive multiorgan damage.^1^^,^^2^^,^^4^^,^^6^, ^7^, ^8^, ^9^, ^10^^a^In individuals without cystinosis, cystinosin, a lysosomal cystine-H^+^ cotransporter, facilitates removal of the amino acid cystine from lysosomes.^1^^,^^2^ The figure depicts the mechanism of disease and cellular effects of cysteamine treatment in patients with cystinosis. ^b^Incidence is likely underestimated.^6^^c^Cystinosis affects males and females in roughly equal numbers and can impact individuals of any race or ethnicity.^11^^d^The 57-kb deletion is the most common *CTNS* variant.^1^^e^A normal WBC cystine level in unaffected individuals is < 0.2 nmol ½ cystine/mg protein for mixed leukocytes.^6^^f^Medical advances include earlier diagnosis, kidney transplantation, and disease-modifying cysteamine treatment.^1^^,^^7^^g^Cysteamine is a cystine-depleting therapy and should be considered for all patients with nephropathic cystinosis, regardless of age or transplantation status.^6^^,^^12^^h^Target granulocyte levels are < 1.9 nmol ½ cystine/mg protein and target mixed leukocyte levels are < 1.0 nmol ½ cystine/mg protein. Tests are not interchangeable.^13^^i^Every year that a patient maintains WBC cystine levels within target range corresponds with roughly 1 year of additional native kidney preservation.^14^ Patients who initiate cysteamine early with sustained adherence also exhibit greater life expectancy and have fewer, less severe extrarenal complications compared with patients who start treatment later, do not adhere to treatment, or are untreated.^10^^,^^15^^j^Not an exhaustive list of complications. ^k^Acute neurologic events may include seizures, stroke, or encephalopathy.^10^^l^Cystinosis has not been shown to cause infertility in women, but significant pregnancy risks may exist.^1^^,^^10^ CKD, chronic kidney disease; GERD, gastroesophageal reflux; IIH, idiopathic intracranial hypertension; MBD, metabolic bone disease; WBC, white blood cell.

Three subtypes of cystinosis have been defined and differ by age at symptom onset, organ involvement, and disease progression.^7^ Accounting for approximately 95% of cases, infantile nephropathic cystinosis is the most severe form of the disease, and the kidneys are the first organs affected as cystine crystalizes in proximal tubular cells.^1^^,^^6^^,^^7^ Patients commonly present with Fanconi syndrome, vomiting, growth failure, and hypophosphatemic rickets from 6 to 12 months of age; and without adequate oral cystine-depleting treatment, advance to kidney failure after the first decade of life.^1^^,^^6^ In addition, patients develop corneal cystine deposits starting at 12 to 18 months of age and require eye drops to minimize photophobia and other ophthalmologic sequelae.^6^^,^^8^ Intermediate or juvenile nephropathic cystinosis is often diagnosed later in adolescence and has a similar but milder course than the infantile form, with less frequent occurrence of impaired growth and Fanconi syndrome. Lastly, the nonnephropathic or ocular subtype only affects the eyes without systemic involvement and rarely presents before adulthood.^8^ More than 140 pathogenic *CTNS* variants have been identified, and though progress has been made, specific genotype-phenotype correlation accounting for the clinical heterogeneity observed in patients within and across cystinosis subtypes has not yet been fully elucidated.^1^^,^^2^

Diagnosis of cystinosis is established via elevated white blood cell cystine levels, genetic testing, and/or presence of pathognomonic corneal cystine crystals on slit-lamp examination.^9^ Ideally, treatment should begin immediately after diagnosis.^7^ Medical management of cystinosis consists of the cystine-depleting agent cysteamine; electrolyte and fluid supplementation for Fanconi syndrome; supportive therapies for chronic kidney disease and other systemic complications; and transplantation and/or dialysis when patients reach kidney failure.^1^^,^^6^ Patient cohort studies have demonstrated that uninterrupted oral cysteamine administration initiated shortly after diagnosis can improve survival, slow kidney function decline, preserve growth, lessen neurocognitive impairment, and delay or prevent extrarenal complications.^15^, ^16^, ^17^, ^18^, ^19^ It has been postulated that patients may retain nearly 1 year of kidney function for every year of sustained cystine depletion within the target range.^14^ However, cysteamine has no effect on tubular damage that is already present, and all patients eventually progress to kidney failure requiring a transplant, even in the context of optimal medication adherence.^17^^,^^19^ Earlier treatment has shifted transplant timing into later adolescence and young adulthood; however, it is currently unknown whether the need for transplantation will be attenuated altogether in patients diagnosed and treated from birth.^6^^,^^17^^,^^19^ Although the allograft helps restore kidney function, it does not correct the underlying metabolic defect in cystinosis, and cystine accumulation continues in nontransplanted tissues.^1^^,^^20^ By adulthood, nearly every organ system is affected (Figure 1).^21^ Therefore, cysteamine must be taken lifelong without interruption, including after kidney transplantation, to limit the onset and severity of extrarenal manifestations, such as metabolic bone disease, hypothyroidism, diabetes, hepatosplenomegaly, myopathy, dysphagia, pulmonary dysfunction, retinopathy, and neurocognitive deficits.^7^^,^^8^^,^^10^^,^^15^^,^^20^ Oral cysteamine formulations are ineffective at depleting corneal cystine deposits, so ophthalmic preparations are needed to reduce progressive photophobia and keratopathy.^1^^,^^10^ Regular monitoring of white blood cell cystine levels is recommended to assess therapeutic cystine control, cysteamine adherence, and need for dosage adjustments.^1^^,^^7^^,^^8^

The advent of cysteamine, in addition to improved diagnostics and advances in kidney transplantation, has dramatically altered the natural history of cystinosis, transforming it from a fatal childhood disorder to a chronic multisystemic condition and extending life expectancy into the 50s and beyond.^1^^,^^7^^,^^22^ Approximately 50% of patients with cystinosis are now adults, necessitating coordinated and comprehensive, yet individualized, management approaches that carry from the pediatric setting across the lifespan.^4^^,^^23^ Because of the rarity of the disease, new challenges have emerged in tandem with evolving patient demographics, including limited awareness and understanding of extrarenal sequelae; fragmented care delivery, particularly among adult nephrology clinics that are not structured to provide multidisciplinary or primary care for complex conditions; disorganized transition to adult clinicians; waning patient and provider engagement; mounting institutional pressure to maximize reimbursement; and minimal evidence to inform lifelong care.^4^^,^^24^, ^25^, ^26^, ^27^ Despite international expert guidance detailing recommendations for the multidisciplinary management of cystinosis, there is still a dearth of published literature on how to best approach establishing disease-specific integrated care teams and/or clinics for rare disorders.^4^^,^^28^^,^^29^ In this review, we summarize key considerations for caring for patients with cystinosis across pediatric and adult settings, outline roles of various providers involved in management, and explore strategies to better support care coordination while fostering long-term patient success.

### Supporting Patients With Cystinosis Across the Continuum of Care

Cystinosis and other rare diseases present a multitude of unique challenges for patients, families, providers, and health systems.^27^^,^^30^ In a survey of 1157 adults living with diverse rare diseases in the US, participants reported that physical symptoms were the most burdensome aspects followed by activity limitations and treatment regimens. Additional difficulties may include diagnostic delays and prognostic uncertainty; limited access to appropriate specialists, medications, and information; clinicians inexperienced in treating cystinosis; insufficient opportunities to develop self-management skills; and stigma and lack of psychosocial support.^24^^,^^30^ Addressing these needs and others across the lifespan is essential to improve rare disease outcomes and quality of life (QOL), which may already be lower than the general US population and those with common chronic illnesses such as arthritis, diabetes, chronic obstructive pulmonary disease, and heart failure.^31^, ^32^, ^33^

#### Multisystemic Complications Requiring Multiple Specialists

As previously mentioned, cystine accumulation has widespread effects, resulting in progressive multisystemic sequelae.^4^ Despite treatment, nearly every organ, including the kidneys, eyes, thyroid, liver, pancreas, gastrointestinal tract, gonads, muscles, bones, lungs, heart, nerves, and brain, is affected to some extent over time; consequently, patients require ongoing surveillance for signs of dysfunction (Figure 1).^4^^,^^10^^,^^21^^,^^34^ In a 2011 Cystinosis Research Network survey, caregivers and pediatric patients reported being most impacted by kidney dysfunction, growth and nutritional deficits, and gastrointestinal complications. Adults agreed that kidney decline was a major concern; however, many expressed concerns about later-onset muscle wasting and feeding or swallowing difficulties.^35^ All complications have the potential to contribute to worsening health status and negatively influence patient QOL; however, myopathy-related dysphagia and pulmonary insufficiency have emerged as significant causes of morbidity and mortality in adulthood.^4^^,^^12^ The incorporation of sensitive discussions about goals of care and participation of palliative/supportive care providers may be important if QOL is significantly affected by these or other major complications such as kidney failure.^34^

The North American Pediatric Renal Trials and Collaborative Studies prospective cohort study confirmed that children and young adults with cystinosis have complex care needs that often entail visits with nonlocal multidisciplinary providers.^36^^,^^37^ To adequately address these needs along with multiorgan complications that extend into adulthood, input is imperative from various specialties and disciplines, such as general/transplant nephrology, genetics, primary care, endocrinology, neurology, gastroenterology, ophthalmology, cardiology, pulmonology, orthopedics, nursing, pharmacy, physical/occupational/speech therapy, nutrition, psychology, and social work (Figure 2).^4^^,^^10^^,^^28^^,^^34^

**Figure 2:**
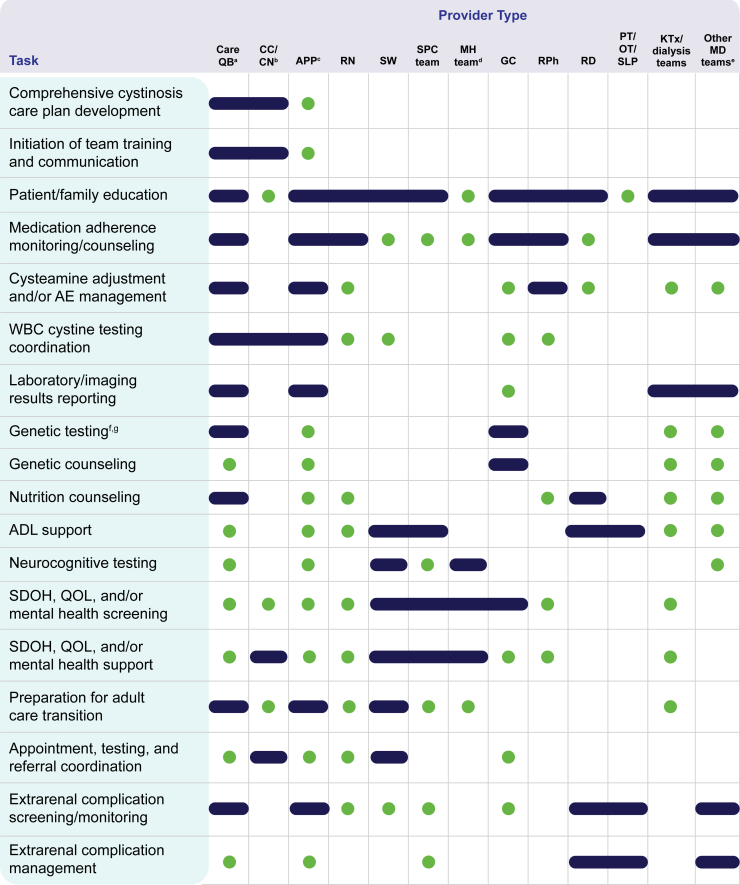
Potential roles and responsibilities for clinical and ancillary support team members in comprehensive cystinosis care. Although clinic or care team workflows and resources vary, this figure depicts ideal core responsibilities (blue dash) and secondary care tasks (green dot) for each provider type.^4^^,^^28^^,^^29^^,^^34^^,^^38^, ^39^, ^40^, ^41^, ^42^, ^43^^a^Primary cystinosis provider or care quarterback can be a nephrologist, clinical or metabolic geneticist, PCP (trained in family medicine, pediatrics, internal medicine, or combined internal medicine and pediatrics [med-peds]) or APP (e.g., NP, PA, CNS) in the listed specialties. ^b^Dedicated care coordinator or navigator could be an APP, nurse, social worker, genetic counselor, medical assistant, or administrative assistant. ^c^Roles listed for APP who is not the primary cystinosis provider or care quarterback. ^d^Mental health providers may include psychiatrists, psychologists, therapists, social workers, and/or counselors. ^e^Other multidisciplinary providers in the extended referral network may include specialists in ophthalmology, neurology, endocrinology, gastroenterology, pulmonology, cardiology, orthopedics, pain management, dermatology, and reproductive health. Involvement will depend on individual patient needs. ^f^Genetic testing also involves assessment of at-risk infants, including siblings of patients with cystinosis and offspring of patients with cystinosis/carriers of *CTNS* variants. ^g^Further genetic analysis outside of initial *CTNS* variant identification may be ordered for patients with cystinosis if additional genetic conditions not already identified via single-gene testing are suspected or to assess potential response or sensitivity to various medications. ADL, activities of daily living; AE, adverse effect; APP, advanced practice provider; CC/CN, care coordinator/care navigator; CNS, clinical nurse specialist; GC, genetic counselor; KTx, kidney transplant; MA, medical assistant; MD, multidisciplinary; MH, mental health; NP, nurse practitioner; OT, occupational therapist; PA, physician associate; PCP, primary care physician; PT, physical therapist; QB, quarterback; QOL, quality of life; RD, dietitian; RN, nurse; RPh, pharmacist; SDOH, social determinants of health; SLP, speech-language pathologist; SPC, supportive palliative care; SW, social worker; WBC, white blood cell.

#### Care Navigation/Continuity and Psychosocial Support Needs

Care navigation and continuity can be particularly daunting to manage in cystinosis because of progressive organ dysfunction that necessitates lifelong multidisciplinary management within increasingly complicated health systems.^4^^,^^24^ Previous studies have shown that children with special health care needs require various forms of medical, social, and academic assistance; and that these services are often fragmented or duplicative, resulting from systemwide inefficiencies and leading to suboptimal care.^38^^,^^39^ Furthermore, children of lower socioeconomic status have greater unmet medical needs while experiencing poorer care quality and coordination.^38^^,^^44^

In addition to juggling several specialists and treatment plans, which can be overwhelming without adequate support, the demands of caring for a child with cystinosis place an immense burden on caregivers.^45^ Parents frequently become the primary coordinators of their child’s care, spending significant amounts of time scheduling clinic visits, often on different days with multidisciplinary providers in separate locations; navigating complex medication regimens; and conveying information to clinicians.^24^^,^^45^, ^46^, ^47^, ^48^ This time toxicity—a concept emerging from cancer care and starting to be applied to chronic and rare diseases—significantly disrupts daily life, impacts wellbeing, and can reduce social support systems and professional success.^49^ In addition, caregivers may face high levels of stress, anxiety, depression, fear about the future, isolation, and sleep deprivation as well as the invisible weight of medical trauma from their child’s diagnostic odyssey and frightening medical experiences.^45^^,^^48^^,^^50^ Limited health system support and challenges, such as ineffective provider communication and collaboration, sparse educational offerings and resources about cystinosis, lack of disease understanding among clinicians, scarcity of services near patients’ homes, prolonged wait periods to see specialists, medical trauma and stigma, and exclusion of patients or families from clinical decisions, can further reduce QOL and collectively erode trust in providers.^51^, ^52^, ^53^, ^54^ Other family members, such as siblings of children with cystinosis, may develop feelings of neglect, resentment, or guilt because of the disproportionate attention given to the affected child and from witnessing the emotional toll on family dynamics.^46^^,^^52^

For pediatric patients with cystinosis, early and repeated exposure to medical interventions, such as gastrostomy tube placement and changes, blood draws, imaging, and hospitalizations, can be highly distressing. Even routine medical events may trigger fear, avoidance, and long-lasting negative associations in settings where care is received. These experiences, particularly when not acknowledged or ameliorated by trauma-informed communication, can lead to symptoms of posttraumatic stress disorder, including flashbacks, nightmares, irritability, emotional withdrawal, and refusal to attend clinic visits. Children may come to associate caregivers and their homes with painful or scary procedures, especially when care is delivered without adequate psychological support. Some pediatric patients internalize a sense of feeling different because of their condition, leading to early struggles with body image, peer relationships, and identity development.^50^

As patients with cystinosis age, the transition from caregiver-supervised pediatric care to adult settings is a particularly vulnerable period that often coincides with other significant life changes, such as entering college or the workforce, moving away from home, and developing more independent relationships, which pose challenges in maintaining care continuity.^55^^,^^56^ This stage can heighten mental health issues, with many emerging adults with cystinosis reporting anxiety, depression, and a sense of isolation.^50^ Patients may also experience greater care fragmentation and difficulty locating adult providers with adequate expertise, leading to gaps in monitoring and management of cystinosis-related sequelae.^57^ Despite guidelines advocating for pediatric to adult care transition programs, insufficient preparation for and execution of the transfer remains commonplace.^4^^,^^55^^,^^57^ A recent survey of 188 general and transplant nephrology centers in the US found that only 20% reported having a dedicated transition clinic, with 32% having neither a transition clinic nor a transition protocol.^58^

Decisions about higher education, employment, place of residence, relationships, and family planning may be dictated by the significant physical burden and management demands of cystinosis.^56^ Families may experience significant economic strain because of direct and indirect medical costs, with fiscal responsibilities shifting to patients as they age.^56^^,^^59^ In a 2011 Cystinosis Research Network survey, emerging adult patients reported particular concerns about aging out of parents’ insurance coverage, securing housing, and going into debt because of medical expenses.^35^ The Cystinosis Research Network’s subsequent 2016 survey found that worrying about paying for housing and medical bills also extends into later adulthood.^60^ These financial difficulties may limit progress toward independence and self-management.^35^ Frequent long-distance travel to receive care may further contribute to indirect medical costs. In addition, patients with rare diseases who live in remote or underserved areas often face challenges in obtaining specialized care, which is more readily available in urban and suburban areas.^59^ Recent data from the North American Pediatric Renal Trials and Collaborative Studies Cystinosis Registry reported that 42% of the 72 children and young adults surveyed live farther than 50 miles from their care center, with another 26% living 20 to 50 miles away, highlighting a significant proportion of patients at risk for inequitable care access.^36^

In adulthood, patients frequently rely on significant others, friends, or family members to help manage their condition, and these care partners may experience emotional strain because of witnessing their loved one’s progressive health decline, resentment about nonadherence, and difficulty balancing care with their own responsibilities and increased caregiving burden.^53^^,^^56^^,^^61^ Patients may experience persistent distress related to their own medical history, including posttraumatic stress disorder symptoms triggered by routine care, anxiety, body image concerns, grief over loss of normalcy, and challenges related to identity and future planning.^50^ Patients often worry and feel guilty about the cumulative impact of their disease and reduced longevity on their families and current or future partners and offspring and may make life decisions with these fears in mind.^48^^,^^56^^,^^62^

The cumulative psychological stressors of managing a rare chronic illness are compounded by a lack of mental health screening and targeted support, despite the known benefits of early intervention.^50^ Addressing barriers to care coordination and continuity, as well as nonclinical and psychosocial challenges, requires integrated support from disciplines such as social work, mental health, genetic counseling, palliative/supportive care, and care navigation or case management to improve overall QOL for those affected (Figure 2). These providers can help alleviate some of the burden on patients and families by providing resources and coping strategies, addressing health disparities, and ensuring access to coordinated care.^34^^,^^48^^,^^50^^,^^56^ Routine screening for medical trauma and referrals to providers for mental health interventions, such as trauma-focused cognitive behavioral, dialectic behavior, acceptance and commitment, or eye movement desensitization and reprocessing therapies and mindfulness-based stress reduction, can address trauma symptoms, increase resilience, and improve care engagement.^50^ Travel assistance programs and telehealth appointments with patients’ regular care teams and/or nonlocal consultant specialists can also facilitate care provision, although these services may contribute their own associated barriers, such as inequities in internet access and regulations that require patients to be physically present in states where providers are licensed during virtual visits.^32^^,^^40^^,^^59^

#### Adherence, Care Engagement, and Self-Management Challenges

Managing numerous medications taken multiple times a day for cystinosis and transplant, including oral and ophthalmic cysteamine, complex immunosuppressive regimens, and other supportive therapies, can be burdensome for patients and families and lead to treatment fatigue, particularly around initial diagnosis, when responsibility shifts from caregivers to patients, and as patients live longer into adulthood.^5^^,^^63^ Despite the known benefits of long-term cysteamine treatment, adherence is challenging for many patients, largely because of its strict dosing regimen and adverse effects such as gastrointestinal distress, body odor, and halitosis; these barriers may be further compounded by psychological factors such as depression, anxiety, or trauma responses.^50^^,^^63^ It is estimated that less than one-third of patients are adherent to the 6-hour dosing schedule with immediate-release cysteamine and that adherence wanes over time in adolescent and adult patients because of difficulty maintaining consistent routines, lack of understanding of the importance of treatment, and burnout as well as desire for independence and normalcy.^63^, ^64^, ^65^ Evidence suggests that adherence may improve with the 12-hour dosing interval permitted by delayed-release formulations of cysteamine, eliminating the need to wake for overnight administration.^66^^,^^67^ In addition to simplifying dosing regimens for cystine-depleting therapy and/or concomitant medications, other strategies to increase adherence include the use of reminders and alarms, ongoing education about the importance of continuous cystine control, implementing measures aimed at reducing side effects, and ensuring medication access.^63^ Although all care team members should be involved in monitoring and counseling on medication adherence, pharmacists, dietitians, social workers, genetic counselors, palliative/supportive care specialists, mental health clinicians, advanced practice providers, and care navigators can help guide patients and families to incorporate complex treatment regimens into daily routines (Figure 2).^4^^,^^34^ Integrating trauma-informed approaches may also reduce care avoidance and foster improved adherence.^50^

Care engagement may be hindered by the limited availability of specialized multidisciplinary providers and the need for complex care coordination. Dovetailing with diminished treatment adherence, patient motivation can be challenging to maintain over the long course of a chronic illness such as cystinosis, and patients/families may experience periods of burnout or detachment, particularly during adolescence and times of transition, which can have major implications on disease progression and allograft survival.^4^^,^^28^^,^^63^ These patterns of avoidance may reflect trauma responses that are not always recognized by providers, particularly when care disengagement is perceived as defiance rather than distress.^50^ Although there is limited data on the impact of long-term, compounded medical trauma on adherence in adults, incompletely developed self-management abilities may exacerbate difficulties in directing care efforts and responsibilities in adulthood.^55^ All care team members should be involved in encouraging care engagement and cultivating self-management because these components acutely and directly impact patient outcomes.^4^^,^^28^^,^^55^ The care team should work to foster a sense of partnership with patients and families by offering support, incorporating their input, and including them in decision-making and goal setting to promote self-efficacy, independence, and active participation. Trauma-informed motivational interviewing, group counseling, and peer mentorship can be effective in helping patients name their fears, reflect on their values, identify their reasons for participating in care, and overcome barriers to adherence and independence.^50^^,^^68^ Referrals to specialized mental health services that address medical trauma, such as cognitive behavioral, dialectic behavior, acceptance and commitment, or eye movement desensitization and reprocessing therapies, may offer needed support for those experiencing trauma-related distress or avoidance. In addition, mindfulness-based stress reduction practices and polyvagal-informed techniques can be used to promote emotional regulation, mitigate care-related anxiety, and foster a sense of agency.^50^

### Enhancing Comprehensive Care Quality and Patient Outcomes in Cystinosis

Coordinating comprehensive care over the course of an individual’s lifetime can pose significant challenges for patients, caregivers, and clinicians, including health system navigation, intra- and interinstitutional provider collaboration, continuity of care during transitions, and access to necessary services. Research has highlighted the critical need to improve care coordination and support for patients with cystinosis and other complex, chronic, and/or rare conditions.^4^^,^^28^^,^^41^

Multidisciplinary care teams and clinics have been most frequently studied in cancer and have become increasingly used in other disorders, including rare multisystemic conditions.^69^ Although it is generally recognized that standardized coordination processes are needed to reduce fragmented care, cost inefficiencies, and adverse health outcomes, there is no consistent definition for care coordination, and consensus on successful and sustainable models is lacking.^38^^,^^44^^,^^70^, ^71^, ^72^ Considering that evidence supporting best practices for coordinated cystinosis management is also limited, we endeavored to conceptualize an integrated multidisciplinary framework that can be scaled up or down, depending on institutional resources and provider availability.

#### Recognizing Factors that Impact Cystinosis Care Delivery

Despite the anticipated benefits of a specialized multidisciplinary cystinosis/rare disease clinic and medical home, we acknowledge that universal adoption is not feasible. Barriers that may hinder full clinic implementation and sustainability include dwindling institutional resources, financial constraints, minimal patient volume, lack of experienced and/or interested providers, noncentralized geographic location, limitations on telehealth, outdated electronic health record systems, and insufficient reimbursement (Supplementary Table S1).^41^^,^^73^ It is our opinion that any elements that can be incorporated into practice, particularly those associated with appointing a primary cystinosis provider or care quarterback, will make a palpable difference in patient and caregivers’ lives.

#### Involving Patients/Families in Shared Decision-making

Patients and caregivers should be viewed as extensions of the multidisciplinary team. Those with active participation in clinical decisions derive greater satisfaction and empowerment from provider interactions, exhibit increased understanding about treatment plans, and have more realistic prognostic expectations, all of which may improve QOL, adherence, and care engagement; and facilitate earlier identification and intervention when complications or nonclinical barriers arise.^74^, ^75^, ^76^ Tailored patient education programs and resources, as well as peer support groups, further promote a positive outlook on cystinosis management and serve as valuable sources of information and connection for patients, families, and providers.^4^^,^^21^^,^^28^ However, it is important to recognize that establishing trust and rapport with patients and families is essential to initiating and maintaining open communication because they may have experienced past medical trauma and providers not believing their symptoms, not listening, or lacking compassion.^30^^,^^50^^,^^77^

#### Designating a Care Quarterback to Reduce Care Fragmentation

To ease the burden of coordinating care on patients, caregivers, and multiple clinicians, a medical home—led by a care quarterback (nephrologist, geneticist, primary care physician, or advanced practice provider) with experience and/or interest in cystinosis—can serve as the central hub for navigating all aspects of patients’ care (Figure 3).^4^^,^^48^^,^^78^ The care quarterback would be responsible for developing care pathways and transition/referral protocols to address cystinosis-related complications, for managing cystine-depleting therapy and routine white blood cell cystine monitoring, for communicating with and educating other specialists added to the team over time, and for liaising with patients and families as a single point of contact (Figure 2).^4^^,^^27^^,^^55^^,^^79^ It is important to note that this provider does not need to be a senior member of the clinical team or an expert in cystinosis but should be open to learning about and staying up to date on the disease and its psychosocial or QOL impacts and be willing to take on the complex and comprehensive care of these patients.^79^

**Figure 3:**
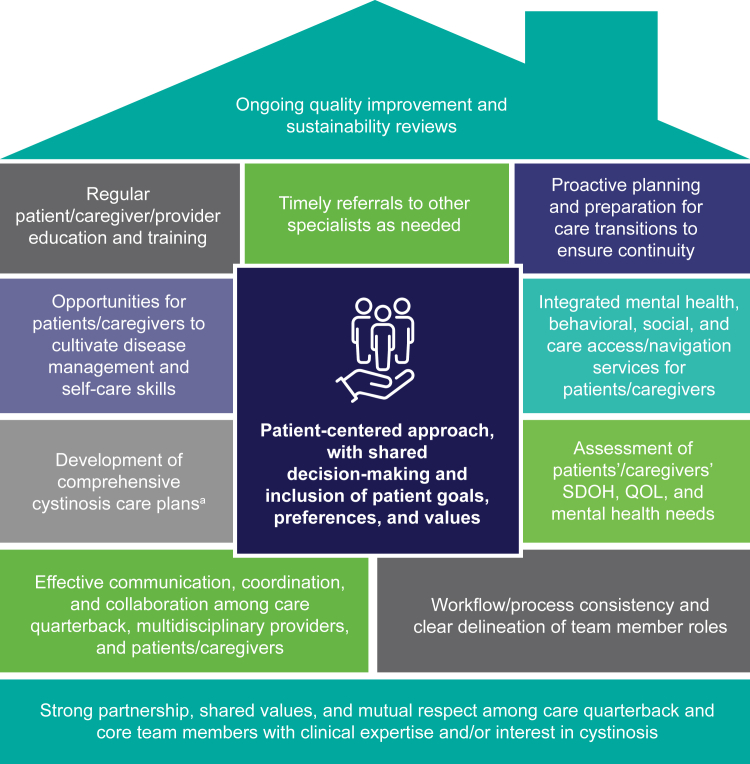
Key elements of integrated management within the medical home for patients with cystinosis.^4^^,^^24^^,^^26^^,^^27^^,^^41^^,^^47^^,^^78^^a^Comprehensive cystinosis care plans should be routinely updated, available within the EHR, and include medical summaries, care transition/emergency action plans, and medication lists. EHR, electronic health record; QOL, quality of life; SDOH, social determinants of health.

#### Establishing a Specialized Multidisciplinary Core Team or Medical Home

Although care coordination is associated with reduced risk of unmet specialty care needs, evidence suggests that the impact is even greater when care is received through a medical home.^44^ This comprehensive approach requires careful planning, partnership, and commitment from providers, institutions, patients, and families (Figure 3). A business plan outlining the clinic or medical home’s mission, goals, and operational structure—including target patient population, projected patient volume, anticipated resource needs, schedule and logistics strategy, roles and responsibilities of multidisciplinary team members, methods for measuring quality improvement, and approach for care coordination and patient centeredness—would help secure buy-in from administrators before the clinic’s inception.^41^^,^^79^ Funding a specialized clinic can be challenging; however, securing institutional support; government, industry, or patient advocacy grants; philanthropic donations; and/or maximized insurance reimbursement can help ensure long-term success and sustainability of the medical home model (Supplementary Table S1).^47^^,^^79^^,^^80^

Following appointment of the care quarterback, further development of the cystinosis medical home can progress in stages, with the next step being creation of a core team of multidisciplinary providers. This team should include representatives from a selected group of specialties with experience and/or interest in rare diseases who are committed to shared goals, mutual trust, effective communication, and measurable processes and outcomes (Figure 3).^41^ Identifying a dedicated patient care coordinator is an important aspect of establishing the medical home because this individual would help run the clinic by supporting appointment scheduling, inbound and outgoing referrals, and information sharing among providers and patients/families, as well as relieving the care quarterback of various administrative and operational tasks.^41^^,^^47^ Care coordinators often develop long-lasting relationships with patients and families as a consistent point of contact, making it easier to identify gaps in care, maintain open channels of communication, and manage issues that may arise in a timely manner.^39^^,^^41^ Because this role requires institutional investment, it may be useful for coordinators to serve a larger population of patients with kidney and/or rare diseases. Other key team members may include advanced practice providers, genetic counselors, pharmacists, social workers, dietitians, and mental health clinicians to assist with streamlining care provision, optimizing medications and nutrition status, addressing psychosocial barriers, and providing patient or family support. Regular team meetings and/or case conferences, either in-person or virtual, led by the care quarterback or care coordinator would ideally be instituted to ensure coordination and execution of management plans as well as ongoing provider education (Table 1).^27^^,^^79^

**Table 1:** Recommendations to improve the patient, family, and provider journey in cystinosis by collaborating across disciplines and specialties

Considerations for comprehensive cystinosis care delivery	
Collaboration	• Engage patients and caregivers or care partners in shared decision-making to enhance medication adherence and care satisfaction/engagement • Schedule regular multidisciplinary team meetings and/or case conferences to discuss patients and provide disease state education
Workflow/Coordination	• Design decision support aids or clinical pathway tools, specialist referral pathways, and individual patient care plans/medical summaries (including relevant medical history and updated medication list) to reduce management variation across care settings • Prepare patients/families and providers for transitions of care by encouraging self-management skill development and outlining procedures for patient handoff, including joint pediatric and adult provider meetings, transition workshops, and/or use of a transition coordinator • When possible or appropriate, schedule in-person provider appointments on the same day and offer follow-up appointments via telehealth platforms to reduce travel/scheduling burden and time toxicity
Outreach	• Communicate with patients/families and providers via shared EHR and secure messaging portals o Consider maintaining a list/database (within or outside EHR) to track patients with cystinosis and ensure regular follow-up, laboratory testing, appointment attendance, and referral follow-through
Assessment^a^	• Consider incorporating standardized assessments into routine practice to identify areas where additional patient/caregiver support is needed o Patient- and/or caregiver-reported outcome measures and QOL: PRO-Kid, PedsQL, TACQOL, QLI, SIP, PNS-RD, MDADI o Mental health: PHQ-9, HADS, WHO-5, DASS-21, Beck Depression Inventory o Transition readiness: TRAQ, TRxANSITION Index, STARx Questionnaire, REALM-Teen, Got Transition® TRA for Youth, Got Transition® TRA for Patients/Caregivers, RTQ o Medication adherence: MMAS, BMQ, ARMS o SDOH: PRAPARE, AHC-HRSN, AAFP Social Needs Screening Tool
Review	• Routinely reevaluate clinic/care team processes to assess for opportunities to improve quality and sustainability and to address evolving patient/family needs

AAFP, American Academy of Family Physicians; AHC-HRSN, Accountable Health Communities Health-Related Social Needs; ARMS, Adherence to Refills and Medications Scale; BMQ, Brief Medication Questionnaire; DASS-21, Depression, Anxiety and Stress Scale 21; EHR, electronic health record; HADS, Hospital Anxiety and Depression Score; MDADI, Monroe Dunaway Anderson Dysphagia Inventory; MMAS, Morisky Medication Adherence Scale; PedsQL, Pediatric Quality of Life Inventory; PHQ-9, Patient Health Questionnaire-9; PNS-RD, Parental Needs Scale for Rare Diseases; PRAPARE, Protocol for Responding to and Assessing Patients’ Assets, Risks, and Experiences; PRO-Kid, Patient-Reported Outcomes Measure in Pediatric Chronic Kidney Disease; QLI, Quality of Life Index; QOL, quality of life; REALM, Rapid Estimate of Adult Literacy in Medicine; RTQ, Readiness for Transition Questionnaire; SDOH, social determinants of health; SIP, Sickness Impact Profile; STARx, Self-management and Transition to Adulthood with Treatment; TACQOL, Netherlands Organization for Applied Scientific Research Academical Medical Center (TNO-AZL) Child Quality of Life; TRA, transition readiness assessment; TRAQ, Transition Readiness Assessment Questionnaire; WHO-5, World Health Organization Five Well-being Index.

a Disease-specific questionnaires for cystinosis have not yet been developed.

#### Developing a Broader Referral Network for Extrarenal Manifestation Management

Expanding beyond the core team to develop an *ad hoc* multidisciplinary network can further address the diverse renal and extrarenal needs of patients with cystinosis. To build this network, the care quarterback and core team should actively engage with colleagues in various specialties, educating them about the disease and importance of lifelong coordinated care. Organizing educational events, grand rounds presentations, and research projects can help foster relationships with other clinicians who may be interested in cystinosis or rare diseases (Table 1).^79^ In addition, collaborating with industry and the several well-established cystinosis advocacy foundations can help with provider identification via established referral lists (Figure 4).^56^^,^^81^

**Figure 4:**
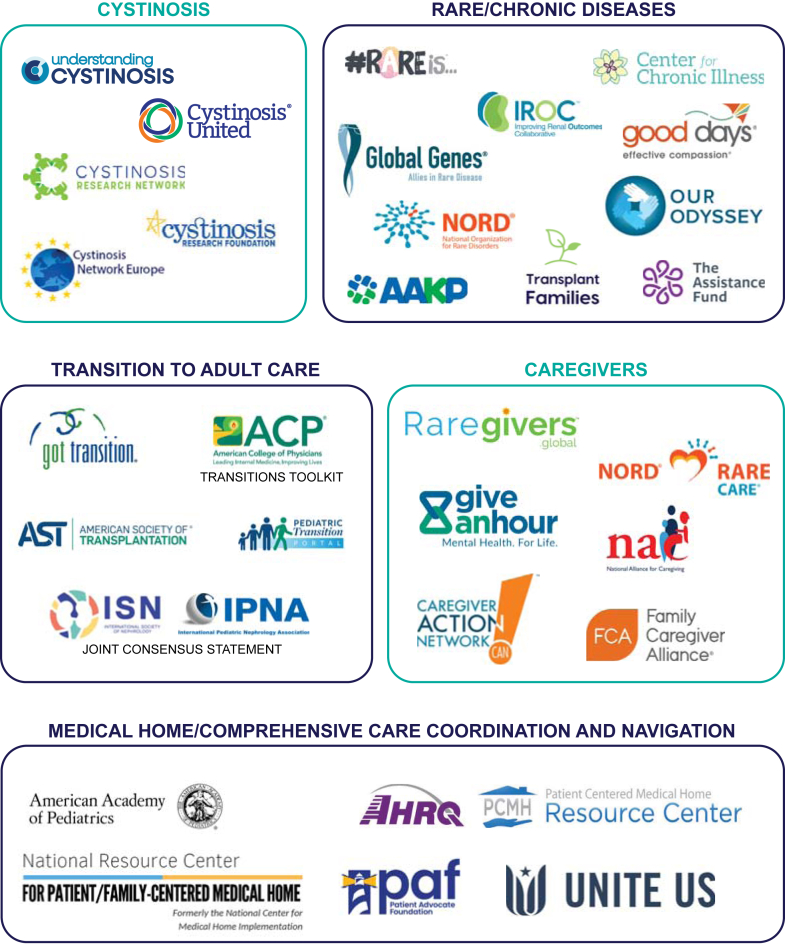
Selected resources for clinicians, patients, and caregivers. AAKP, American Association of Kidney Patients; AHRQ, Agency for Healthcare Research and Quality.

#### Optimizing Care Coordination, Quality, and Communication

In addition to approaches previously mentioned, several strategies to enhance care delivery, quality, and continuity have been proposed, including use of medical summaries and comprehensive care/emergency action plans that clearly delineate provider roles and summarize relevant clinical history, distribution of cystinosis-specific fact sheets to educate clinicians and onboard new team members, and expansion of the medical home to include patients with other multisystemic rare diseases in care settings with limited resources and/or low patient volumes. Furthermore, communication and care coordination could be improved by establishing 1- or 2-day cystinosis clinics during which multiple provider appointments and examinations are scheduled to reduce patient or caregiver travel burden and time toxicity; and by facilitating real-time information exchange among patients, caregivers, and multidisciplinary providers via secure messaging/telehealth platforms and shared electronic health record systems (Table 1).^4^^,^^41^^,^^42^^,^^55^ Incorporation of patient/family feedback can also strengthen the care team’s continuous quality improvement efforts.^27^

#### Facilitating Effective Care Transitions

A structured transition plan from pediatric to adult settings is important to ensure continuity of care and should begin early in adolescence, with gradual transfer of responsibilities from caregivers to patients for medication management, appointment scheduling, involvement in care decisions, and communication with providers.^55^^,^^57^ Readiness assessments may be useful to evaluate patient autonomy, responsibility, emotional stability, cognitive maturity, overall health literacy, disease understanding, and treatment adherence; and to determine optimal and individualized transfer timing.^48^^,^^54^^,^^82^ It is also important to set realistic expectations for patients and caregivers about care provision in adult settings, particularly regarding coordination, continuity, and resource limitations across multiple specialists and/or health systems.^57^ Pediatric care teams typically act as a single source for a variety of renal and extrarenal services and have longstanding relationships with patients. As such, they should work closely with adult providers—who may be new to managing patients with cystinosis—to ensure a smooth handoff and to help establish patient trust in adult teams.^48^^,^^57^ Although this coordination is underutilized, it can involve joint pediatric-adult clinic visits, educational workshops, development of a comprehensive transition summary, periods of regular communication between pediatric and adult providers, transfer of medical records to new clinicians, assistance of a transition navigator, a dedicated transition clinic, and/or procedures to capture missed appointments and ensure that patients are not lost to follow-up.^4^^,^^48^^,^^55^^,^^57^^,^^63^ Because transition planning and execution are increasingly recognized as priorities for health care improvement, resources are available through several professional and patient associations (Figure 4).^56^^,^^82^ It is worth noting that some disciplines, such as genetics, combined internal medicine and pediatrics (med-peds), and family medicine may retain patients throughout the lifespan. In these cases, a traditional transition to adult care is not needed; however, clinicians should still strive to ensure that patients are able to more independently manage their care in adulthood.^4^

Patients with cystinosis may experience other transitions of care at different points along their disease journey, including to/from dialysis and transplant teams; to/from inpatient units; and to new providers because of relocation, retirement, insurance changes, or need to involve additional specialists.^38^ The cystinosis care quarterback or medical home core team should explore the development of protocols for communicating with teams in other settings or institutions, providing access to relevant medical history and disease information, ensuring ongoing monitoring and management of extrarenal complications, and following up after transition (Figure 3).^24^^,^^43^

#### Tailoring Care to Individual Patient/Family Needs Across the Lifespan

High-quality care provides patients and families with access to resources that address their unique and evolving needs at the right time in the right place. Because many individuals with cystinosis do not live near a major medical center, patients or caregivers may opt for care plans to be implemented by local providers whenever possible.^41^ In addition, patients may require or prefer varying levels of education and coordination assistance across the continuum of care, ranging from simply detailing where and how to obtain information to managing integrated services on their behalf.^38^^,^^41^^,^^83^ Individualized patient-centered care can be further achieved through comprehensive medical, psychosocial, educational, and family needs assessments; regular management plan reviews and updates; flexible appointment scheduling and care delivery options; connection to specific community resources; and age-appropriate services (Figure 3).^27^^,^^38^^,^^83^ In pediatric settings, early psychosocial support, family education, and structured transition planning can encourage resilience and self-management skills. In adulthood, approaches focused on mental health, vocational support, and long-term disease management are crucial for sustained independence and QOL.^38^

### Conclusion

Individuals with cystinosis have improved health outcomes and prolonged survival, resulting from advances in diagnostics and medical management over the past 5 decades.^7^^,^^84^ A vibrant cohort of adults has grown from this success; however, new challenges have emerged, including later-onset extrarenal comorbidities and care delivery that varies by health system and is often fragmented.

Although multidisciplinary rare disease programs can demand significant amounts of time, effort, and institutional investment to develop, collaborative teams are imperative to address the multisystemic manifestations of cystinosis and reduce the burden of care coordination on patients and families. Proposed medical home models use a nephrologist, primary care physician, geneticist, or advanced practice provider in the vital role of care quarterback, surrounded by a core team and expanded network of other specialties and disciplines, ensuring patients receive comprehensive care tailored to their complex individual needs. Coordinated provider efforts may not only enhance care quality and continuity but also assist patients and families in navigating the peaks and valleys of living with cystinosis.

Considering that the time and resources needed for the proposed multidisciplinary cystinosis care model extend beyond those typically reimbursed in current fee-for-service models, we advocate for the development of dedicated billing codes for chronic, rare disease management that would encompass care coordination as well as services usually covered under facility fees, such as those provided by social work, pharmacy, nutrition, genetic counseling, mental health, and care navigation. These additional codes would incentivize collaborative longitudinal care and enable providers to deliver nonmedical services that are essential for high-needs, high-cost patients, such as those with cystinosis. Health policy experts note that flexibility to pay for selective, high-value social services is critical because these supports are often as impactful as medical interventions for individuals with chronic and complex conditions.^85^

## Disclosure

MHD, CG, LG, PCG, JLH, FJK, AL, LSM, BS, and JJZ have received honoraria from Amgen Inc (formerly Horizon Therapeutics plc) for past consulting/advisory activities. BS and JJZ are members of Amgen Inc’s Speaker Bureau. CG and LSM are recipients of a grant from Amgen Inc for a transition clinic program, which was paid to Northwestern Medicine. EGA has received funding for travel from Moderna and honoraria from Asklepios BioPharmaceuticals Inc; and serves on the Scientific Advisory Board for the TANGO2 Research Foundation. PG has served as a consultant for Genentech and Recordati. KJS declared no competing interests.

## Supplementary Material

Supplementary File (PDF) **Table S1.** Considerations for maximizing reimbursement for comprehensive cystinosis management.

## Footnotes

Supplementary File (PDF)
**Table S1.** Considerations for maximizing reimbursement for comprehensive cystinosis management.
