# Supplementary material for: Improving Lifelong Comprehensive Care Coordination in Nephropathic Cystinosis: Multidisciplinary Perspectives
Source: Kidney Int Rep. 2025 Dec 18;11(3):103735. doi: 10.1016/j.ekir.2025.103735 (PMC12828517; doi:10.1016/j.ekir.2025.103735)
Supplement: Supplementary File (PDF) — Table S1. Considerations for maximizing reimbursement for comprehensive cystinosis management. [file mmc1.pdf]

SUPPLEMENTARY TABLE S1. Considerations for maximizing reimbursement for comprehensive cystinosis management.[a] In the United States, effective billing for cystinosis care requires the use of appropriate ICD,[b] CPT,[c] and/or HCPCS[d] codes to encompass the extent of services provided for given diagnoses. As billing and coding guidance frequently changes, refer to the organizations under the *Selected resources* section for the most up-to-date information.

**Billing and reimbursement considerations**		
**Outpatient services that may be billable**	**Description**	**Notes and caveats**
**Prolonged patient visit**	Evaluation and management appointment for a patient that exceeds a certain time threshold defined by insurance provider	For split/shared services during a visit in the facility setting that is performed by 2 providers who are in the same group, only the provider (physician, NP, PA, or CNS) who performed the substantive portion of the visit and/or medical decision-making should bill for the visit
**Chronic care management, including care coordination and case management**	Comprehensive care that may include management of chronic conditions, referrals to other providers, medication management, facilitation of transitions of care, patient needs assessment, and ongoing review of patient status and the care plan	Patient must meet a certain threshold for number of chronic conditions depending on insurance provider<br><br>Patient consent to participate in the program is typically required<br><br>Chronic care management services, including care coordination and case management activities, must be documented in a personalized comprehensive care plan in the EHR<br><br>Complexity of care and medical decision-making may dictate billing codes and reimbursement amounts<br><br>Patient must have 24/7 access to a member of the care team for urgent needs as well as availability of multiple methods of communication to reach providers<br><br>A set amount of clinical staff time per calendar month, as dictated by the insurance provider, must be spent on non–face-to-face chronic care management services directed by the billing provider (physician, NP, PA, or CNS)<br><br>Only one provider who assumes the care management role can bill for chronic care management services to a given patient per calendar month
**Telehealth visit**	Clinical appointments provided via audio-only or audio-video platforms	Occasional in-person visits may be required depending on insurance provider
**Remote physiological monitoring**	Technology to monitor and analyze a patient's physiological metrics for an acute or chronic condition	Only one provider (physician, NP, PA, or CNS) can bill for remote physiological monitoring per patient within a given period defined by insurance provider<br><br>Remote physiological monitoring and remote therapeutic monitoring cannot be billed together<br><br>Remote physiological monitoring and remote therapeutic monitoring, but not both, may be billed concurrently with the following care management services for the same patient as long as time and effort are not counted twice: chronic care management, transitional care management, behavioral health integration, principal care management, chronic pain management<br><br>Physiological data must be electronically collected and automatically uploaded to a secure location where the data can be available for analysis and interpretation by the billing provider

**Remote therapeutic monitoring**	Monitoring and analysis of non-physiological data, often self-reported, related to therapeutic treatment adherence and response	Only one provider (physician, NP, PA, or CNS) can bill for remote physiological monitoring per patient within a given period defined by insurance provider<br><br>Remote physiological monitoring and remote therapeutic monitoring cannot be billed together<br><br>Remote physiological monitoring and remote therapeutic monitoring, but not both, may be billed concurrently with the following care management services for the same patient as long as time and effort are not counted twice: chronic care management, transitional care management, behavioral health integration, principal care management, chronic pain management
**Patient education**	Education and training for patient self-management for a certain time threshold defined by insurance provider	Face-to-face patient encounter may include caregiver/family members and can include 1-on-1 or group sessions with multiple patients<br><br>Must use a standardized curriculum that is consistent with guidelines or standards established or recognized by a health care professional society or association<br><br>Patient must be present and actively engaged in the education and training
**Medical team conference**	Multidisciplinary team meeting to discuss a patient's care plan for a certain time threshold defined by insurance provider	A minimum number of providers from different specialties or disciplines, as defined by insurance provider, who provide direct care to a patient must participate in the team conference<br><br>Reporting participants must be present for the entire team conference<br><br>Reporting participants must have performed face-to-face evaluations or treatment of the patient, independent of any team conference, within a defined time period prior to the conference, as specified by insurance provider<br><br>Patient and/or caregiver presence is not required
**Interprofessional consultation**	Assessment and management service in which a patient's treating provider requests an opinion and/or treatment advice from a consultant with special expertise via telephone, internet, or EHR to assist the treating provider in diagnosis and/or management of the patient's condition	Does not require face-to-face interaction between the patient and consultant
**Health behavior assessment and intervention**	Services to identify and address psychological, behavioral, emotional, cognitive, and social factors that can impact physical health and treatment adherence	Assessment and provision of services related to physical health, such as adherence to medical treatment, symptom management, health-promoting behaviors, health-related risk-taking behaviors, and adjustment to physical illness<br><br>Codes require a primary physical health diagnosis (ICD) and cannot be used for mental health/psychotherapy services<br><br>Billing providers include clinical social workers, marriage and family therapists, mental health counselors, and clinical psychologists
**SDOH risk assessment and community health integration**	Services to identify and address known or suspected social needs that affect the diagnosis and/or treatment of medical problems	SDOH risk assessment must be reported billing provider (physician, NP, PA, or CNS) in conjunction with an evaluation and management visit, behavioral health visit, or annual wellness visit<br><br>Community health integration services connect patients with appropriate clinical and social support resources and may be provided monthly following an initiating evaluation and management visit
**Other services**	**Medication therapy management**	Face-to-face patient encounter during which a pharmacist reviews medication regimens and provides counseling to improve adherence
	**Genetic counseling**	Total time provided by a medical geneticist and/or genetic counselor on the date of the patient encounter, as permitted by jurisdiction

	**Nutrition counseling**	Face-to-face patient encounter during which a registered dietitian or nutritionist provides recommendations on dietary intake to improve health
	**Advance care planning**	Face-to-face encounter during which a billing provider (physician, NP, PA, or CNS) and a patient to discuss the patient's health care wishes if they become unable to make decisions about their care. As part of this discussion, a provider may talk about advance directives with or without completing legal forms<br><br>Service can occur as a stand-alone visit or as part of an evaluation and management visit
	**Services not covered under other categories**	Institutions may be able to charge facility fees to cover services and/or provider time that are not billable through other channels or billing codes

Selected resources

Association/ Resource	Website(s)
**WHO – ICD**	www.who.int/standards/classifications/classification-of-diseases
**AMA – CPT**	www.ama-assn.org/practice-management/cpt
**CMS – ICD-10 and List of CPT/HCPCS Codes**	www.cms.gov/medicare/coding-billing/icd-10-codes<br>www.cms.gov/medicare/regulations-guidance/physician-self-referral/list-cpt-hcpcs-codes
**AAFP – Medical Billing & Coding**	www.aafp.org/family-physician/practice-and-career/getting-paid/coding.html
**AAP – Coding Fact Sheets**	www.aap.org/en/practice-management/child-health-finance-payment-strategy/coding-and-valuation/coding-fact-sheets
**ACP – Coding for Clinicians**	www.acponline.org/practice-career/business-resources/coding
**RPA – Coding and Billing**	www.renalmd.org/page/coding-billing

[a]Not an exhaustive list of all billable services provided to patients with cystinosis. Reimbursement will vary by clinical situation, provider type, insurance carrier, and country.
[b]ICD codes are maintained by WHO and identify disease and injury diagnoses. The ICD-10 and ICD-11 diagnostic codes for cystinosis are E72.04 and EC60.1, respectively.
[c]CPT codes are maintained by the AMA and identify medical services and procedures.
[d]HCPCS codes are maintained by CMS and identify medical procedures, services, and supplies not included in CPT.

AAFP, American Academy of Family Medicine; AAP, American Academy of Pediatrics; ACP, American College of Physicians; AMA, American Medical Association; CMS, Centers for Medicare & Medicaid Services; CNS, clinical nurse specialist; CPT, Current Procedural Terminology; EHR, electronic medical record; HCPCS, Healthcare Common Procedure Coding System; ICD, International Classification of Diseases; ICD-10, International Classification of Diseases, Tenth Revision; ICD-11, International Classification of Diseases, Eleventh Revision; NP, nurse practitioner; PA, physician associate; RPA, Renal Physicians Association; SDOH, social determinants of health; WHO, World Health Organization.
